# Two decades of majoritarian hegemony and minority subordination within Indonesian religious governance amidst polarization

**DOI:** 10.3389/fsoc.2026.1874907

**Published:** 2026-09-09

**Authors:** Pundra Rengga Andhita, Evi Novianti, Suwandi Sumartias, Agus Rahmat

**Affiliations:** Faculty of Communication Sciences, Padjadjaran University, Bandung, Indonesia

**Keywords:** houses of worship permits, Indonesian religious governance, juridical resonance, majoritarian hegemony, minority subordination

## Abstract

This study examines the socio legal governance and structural exclusion embedded within the implementation of the 2006 Joint Ministerial Decree (PBM 2006) in Indonesia over the past two decades. Using a qualitative critical paradigm with an extensive multilocus fieldwork approach across multiple urban and rural regions, this research investigates how localized administrative discretion shapes civic space. The empirical data are sorted through a disciplined sequential progression starting with a descriptive textual classification before transitioning to independent social theories. The results indicate that the vague lexical design of PBM 2006, particularly regarding neighborhood supporter metrics and executive discretion, converts administrative procedures into tools of selective exclusion. The discussion demonstrates how street level bureaucrats under localized populist pressure systematically employ their discretionary power to enforce administrative delay and spatial containment, a process conceptualized as bureaucratic violence. This institutional erasure is further exacerbated by underlying material conflicts, including economic inequality and property disputes, proving that localized frictions are driven by structural pathologies rather than pure theological clashes. Crucially, the rigid demographical metrics also trigger a juridical resonance that sparks internal division within the dominant majority itself. This study concludes that a shift from approval based permission models toward technical city planning notification systems is imperative to secure a transformative cultural citizenship and guarantee sustainable democratic governance.

## 1 Introduction

The consolidation of post authoritarian democracies often exposes a critical friction where universal human rights guarantees confront the localized bureaucratization of faith. While the roots of Indonesian religious statecraft trace back to the centralized ‘godly nationalism’ and structured harmony parameters of the New Order era, the landscape has undergone a radical, more exclusionary transformation over the past two decades driven by rising polarization (Menchik, 2014). Moving away from the traditional, state regulated majoritarian social equilibrium of the 1969 Joint Ministerial Decree, post 1998 democratization paradoxically gave rise to majoritarian hegemony, a structural condition influenced, among other factors, by transnational fundamentalist movements to structurally reshape the communal identity of the localized majority. In the contemporary context, this universal majoritarian phenomenon manifests concretely through the ideological shifting of this dominant group, wherein historically inclusive practices are increasingly challenged by exclusive doctrines. From several international perspectives, this localized dominant majority socioreligiously corresponds to the mainstream religious entity, which demographically constitutes the dominant population in the nation. As argued by Bruinessen (2011), post authoritarian developments have significantly altered the global image and internal perception of this dominant communal group, shifting them away from a historically tolerant and compromise inclined posture toward a widespread “conservative turn” within the country’s sociopolitical landscape. This internal reorientation is heavily tied to the influx of transnational fundamentalist currents, which systematically export intolerance and radical doctrines into the localized sphere, thereby diminishing the indigenous, peaceful, and culturally adaptive character of the local majority. Consequently, these transnational currents have reshaped identities by layering literal, rigid, and puritanical doctrines over traditional, indigenous practices that were historically characterized by syncretic, inclusive, and adaptive cultural elements.

Consequently, this fundamentalist shift has reframed the mindsets of local communal groups, generating anxieties that systematically polarize society into a rigid dichotomy of ‘insiders’ versus ‘threatening outsiders’. Beyond traditional cultures of pluralistic coexistence, these shifting identity dynamics do not merely exist as theological debates; they actively contribute to a systematic reorganization of physical boundaries. As Roggeband and Krizsán (2021) demonstrate, “closure is in fact a selective mechanism: governments attempt to reorganize civic space through a dual process of selective in and exclusion of civil society organizations.” Within a majoritarian landscape, this technocratic closure of civic space is explicitly translated into the regulation of physical territory, turning the administrative permit process into a tool of spatial containment. This analytical lens exposes how communal anxieties dominate the public sphere, leading to direct social polarization and horizontal actions such as the administrative blocking and systematic sealing of minority houses of worship. Nevertheless, macro sociological evaluation confirms that when a national religious majority is positioned as a localized minority within regions dominated by another faith, they also confront similar patterns of obstruction, social exclusion, and occasional violence when attempting to construct their places of worship. This institutionalized homogenization is fundamentally driven by a shifting political landscape where, as Hurriyah (2020) notes, “the deterioration of religious freedom in Indonesia is linked to the increased influence and ability of religious conservative actors to dominate religious discourse and simultaneously influence government policies… resulting in persistent religious discrimination against minoritized groups”. Ultimately, “this structural approach legitimizes the majoritarian control over public spaces, including the legal verification and spatial placement of minority houses of worship” (Menchik, 2016).

Within the global landscape of social stratification, the structural neutralization of minoritized entities frequently operates through the deployment of bureaucratic obstruction (Heckert, 2020), wherein technocratic state protocols are systematically utilized to enforce long term spatial containment (Rhoads et al., 2025). In the contemporary context, this universal majoritarian mechanism manifests concretely through the technical parameters of the Joint Ministerial Decree of the Ministry of Religious Affairs and the Ministry of Home Affairs Nos. 9 and 8 of 2006, hereafter referred to as PBM 2006. This regulation was designed as an update to the Joint Decree of the Two Ministers of 1969, which served as the legal basis for establishing houses of worship during the New Order era but faced structural challenges in adapting to the dynamics of the reformation and decentralization eras. PBM 2006 was introduced as a technical guide for local governments to maintain religious harmony, including the regulation of procedures for establishing houses of worship through requirements for user and local community support, as well as recommendations from state formed interfaith institutions. This localized regulatory imposition constructs a profound normative friction with international human rights standards, which dictate that structural manifestations of faith should not be subjected to majoritarian concessions. Article 18 of the ICCPR explicitly affirms that everyone has the right to freedom of religion, which includes the freedom to manifest one’s religion or belief in worship, observance, practice, and teaching, either individually or in community with others, and in public or private. This mandate strictly guarantees the freedom of every individual to practice their religion or belief, where restrictions are only justified if prescribed by law and necessary to protect public order or the fundamental rights of others. In practice, the administrative procedures of PBM 2006 often shift the protection of the right to establish houses of worship into a barrier for minority groups seeking permits (Crouch, 2010; Kaur and Hasan, 2026).

Alongside its role as an administrative instrument to maintain civic order, this technocratic framework within its sociological dynamics tends to reproduce majoritarian hegemony. Longitudinal monitoring data compiled by the SETARA Institute, a prominent independent Indonesian non governmental organization specializing in democratic liberties and religious freedom advocacy, across a two decade spectrum from 2007 to 2025, illuminates the systematic expansion of spatial containment under PBM 2006. Through an independent cumulative calculation synthesized and compiled from the raw data sheets within the institute’s periodic annual assessment reports spanning from 2007 to 2025, this study unmasks a total accumulative threshold of 589 actions obstructing houses of worship (Naipospos et al., 2007; Hasani, 2009; SETARA Institute, 2010, 2014, 2018a,b; Satriani et al., 2019; Sigit and Hasani, 2021; SETARA Institute, 2022, 2024; Kaur and Hasan, 2026). Ultimately, this multi year dataset confirms that infrastructure rejections operate as an exclusionary administrative filter, allowing majoritarian states to maintain dominant territorial aesthetics under the framework of routine regulatory compliance while systematically shrinking local coexistence. Despite various technocratic compromises, the tension between constitutional guarantees of religious freedom and administrative constraints at the local level remains a central point of contention. The urgency of this examination is further underscored by the role of regulation as a persistent site of struggle, where its implementation often exacerbates social and political tensions, especially among minorities (Crouch, 2010). Local regulations frequently deviate from constitutional mandates due to political power struggles and legal inconsistencies (Badurdeen et al., 2026). Ultimately, these religious conflicts are not merely a product of spontaneous fundamentalism; they are systematically triggered by the enforcement of state policies and regulations with restrictive impacts on minority groups, reinforcing their vulnerable position within society (Khanif, 2022). While normatively no belief system justifies intolerance, societies continue to witness deep seated diversity conflicts that culminate in the restriction and destruction of houses of worship (Muhajarah and Soebahar, 2024). Empirically, this dynamic highlights how “the post Reformasi era has witnessed escalating tensions between the dominant majority and the affected minority, with persistent discrimination further compounded by the government’s reluctance to offer workable solutions” (Perkasa and Leonard, 2023).

This localized regulatory friction has long garnered serious attention within the international academic community, which intensively highlights how contemporary dynamics reflect global challenges in protecting human rights and religious freedom. Global scholars have begun to dissect how a country’s demographic landscape, with its massive dominant majority, reinforces a politics of recognition that is often exclusive (Hamayotsu, 2014). This phenomenon confirms the existence of systemic mechanisms legalized through bureaucratic procedures, where legal instruments are utilized to restrict the spatial presence of vulnerable communities. Internationally, administrative approval requirements for establishing houses of worship are viewed as effectively granting a veto right to the local majority (Marshall, 2018), while numerical thresholds normalize the expectation that minority claims must be subordinated to dominant communal sentiments (Pedersen, 2016). Driven by these rigid paradigms, dominant groups eventually act as powerful moral arbiters (Sealy et al., 2022), generating a majoritarian politics where standardized boundaries define national membership (Duile and Bens, 2017). Crucially, this exclusionary push contributes to a deeper cultural homogenization and acute polarization that dismantles traditional, pluralistic cultures of coexistence. Resistance to houses of worship is carried out not only across different faiths but also by internal factions with differing views (Zakiyah et al., 2025). This reality demonstrates that the issue of houses of worship is a manifestation of deep structural tensions that systematically polarize society beyond traditional boundaries. Such complex sociopolitical dynamics demand a shift in analytical focus, as previous research has focused on the causal forces of conflict rather than deconstructing specific aspects, such as regulation, that could actually facilitate social cohesion (Robinson, 2016).

Although this regulatory framework has not undergone formal revisions since its enactment, current policy discussions face limitations in fully accommodating the dynamic social realities on the ground. This misalignment is crucial because the contemporary social phenomena arising from the implementation of rules, ranging from minority community negotiation strategies to patterns of local resistance, can provide valuable lessons for the global community in understanding how communal relations are managed within a highly diverse democratic state. Therefore, this phenomenon needs to be elevated more broadly into the global academic space to challenge and enrich universal theories regarding minority protection and religious freedom. By bringing this discourse to an international level, local experiences can offer new perspectives on how democratic, majoritarian dominant nations balance the demands for communal harmony with individual rights guaranteed by international law. Furthermore, this examination aims to reconstruct a more inclusive governance model capable of mitigating the acute socioreligious polarization and cultural homogenization that are often unaccommodated within generalized global perspectives.

The technocratic failure to mitigate these tensions reveals deep ambiguities within the regulatory text itself, necessitating an interdisciplinary dissection. Because these legislative ambiguities are fundamentally embedded within asymmetric structures of power and dominant public discourses, this study establishes a critical intersection by intertwining sociology, legal science, and Critical Discourse Analysis (CDA). Within this framework, CDA is deployed not merely to examine textual syntax, but to structurally deconstruct how authoritative state texts operate as ideological instruments that institutionalize cultural uniformity, thereby establishing the necessary empirical and linguistic foundations to reconstruct a more inclusive alternative text, restore civic sovereignty, and transform asymmetric power relations to facilitate social cohesion. The precision of this integrated framework is required because the law is often trapped in a conventional ambition to eliminate textual ambiguity through the accumulation of technical data, whereas ambiguity is a fundamental feature of language (Choi, 2024). The relationship between communication and law is crucial because legal rules are essentially acts of power communication, given that legal language inherently possesses power behind discourse, which is explicitly reflected through specific lexical choices that subordinate minoritized identities (Khaleel et al., 2023). Within this interdisciplinary realm, CDA is uniquely capable of revealing what is systematically neglected by the text, and how legal texts transform and evaluate the social reality they represent (Van Leeuwen, 2018). To mitigate tensions in the relationship between the state and society, the deployment of discourse modes that prioritize adequate explanation is vital to achieving a just balance in law enforcement (Wu and Sun, 2019). This approach is even more pertinent considering that language is never neutral, even when legal products attempt to project an impression of absolute neutrality; the choice of words and grammar actively represent actors, events, priorities, and responsibilities (Cheng and Machin, 2023). Ultimately, this legal communication paradigm emerges as a powerful critical socio legal intervention, offering alternative interpretive models to untangle how state legislative texts function as instruments of minoritarian subjection while providing global sociology with new conceptual foundations for reconstructing inclusive civic spaces within deeply polarized democratic societies.

## 2 Methods

### 2.1 Research design

This study adopts a qualitative critical paradigm to dissect the socio legal governance and structural exclusion embedded within the implementation of the PBM 2006. Moving beyond a purely descriptive approach, this research implements a sequential progression where structural patterns identified through Norman Faircloughs CDA are positioned as preliminary classificatory findings in the Results section (Fairclough, 1992; 2003; 2009; 2013a; 2013b). Subsequently, these findings are evaluated in the Discussion section using independent social theories principally Michael Lipskys street level bureaucracy and the modalities of bureaucratic violence to generate robust causal explanations.

### 2.2 Sampling strategy

To capture structural variations in the field and mitigate geographical bias, this research employs a purposive sampling strategy executed within a multilocus fieldwork framework. The primary empirical investigation focuses on six distinct regions across Java: DKI Jakarta, Bandung, Bekasi, Banjarnegara, Banyumas, and Cilacap. These loci were purposively selected to contrast high intensity spatial religious conflict zones with stable administrative environments. Informants were selected based on their operational proximity to house of worship permit disputes until data saturation was achieved. To ensure a balanced institutional matrix, the sample comprises 13 key informants: three state apparatus representatives (two officials from the Ministry of Religious Affairs and one from the Ministry of Home Affairs), one expert analyst, and nine figures from religious organizations representing the six officially recognized faiths, all of whom possess direct operational ties to the investigated loci.

### 2.3 Data collection procedures

Primary and secondary data were gathered through three integrated interdisciplinary procedures conducted from March 2025 to March 2026. This specific 12 month timeframe was designated to capture the shifting sociocultural dynamics and the longitudinal continuity of administrative barriers across the primary loci. By deploying an interdisciplinary triangulation framework, this data collection trajectory was structurally calibrated to bridge the analytical gap between macro level textual legislation often framed through technocratic ideals and the micro level socio legal realities experienced by grassroots actors during localized permit disputes.

#### 2.3.1 In depth interviews

Semi structured qualitative interviews were conducted with the 13 key informants based on the criterion of their direct operational engagement with PBM 2006. The interview protocols were specifically designed to capture the discursive asymmetry between macro level technocratic narratives and micro level grassroots realities. By juxtaposing the top down perspectives of central state officials who project an idealized image of systemic harmony on paper with the lived experiences of religious leaders at the grassroots within specific loci, the interviews systematically expose the latent socio legal frictions and structural discrepancies that occur during localized permit disputes.

#### 2.3.2 Observation

Direct socio legal field observations were conducted across designated public spaces within the six primary loci. This procedure prioritizes the researcher’s direct, on site assessment of specific geographic trajectories, spatial configurations, and physical environments directly tied to the empirical boundaries of the study. Crucially, to capture nuanced micro sociological dynamics, the researcher engaged in ethnographical immersion by examining the socioeconomic activities shared between house of worship leaders and local communities. This approach serves as a methodological instrument to capture localized material interactions and class based spaces, thereby enabling the framework to evaluate competing alternative explanations, such as economic inequality, directly at the grassroots level.

#### 2.3.3 Document review

A regulatory document review was performed on the policy framework of PBM 2006, focusing specifically on the articles indicated to cause polemics based on the findings from the in depth interviews. These specific policy nodes were textually cross examined by the researcher with empirical monitoring reports from the SETARA Institute, investigative findings from the National Commission on Human Rights (Komnas HAM), official datasets from the Ministry of Religious Affairs, verified domestic media coverage, and international journal publications to map the structural consistency of administrative barriers across different regions while balancing the study’s baseline against validated external benchmarks.

### 2.4 Data analysis

Data reduction and analysis follow a highly disciplined trajectory that rigorously separates empirical textual sorting from explanatory theoretical analysis. In the initial phase, confined exclusively to the Results section, Norman Fairclough’s three dimensional framework is deployed strictly and proportionally as a preliminary analytical tool to deconstruct the selected regulations. This critical framework operationalizes the textual register to map lexical ambiguities, the discursive register to categorize the technocratic deployment of ‘harmony’, and the sociocultural register to group these findings within broader societal constraints. Crucially, once these structural anomalies within the text are dismantled, the descriptive registers of Fairclough are set aside. The analysis then transitions completely toward a deeper explanatory phase in the Discussion section. Within this section, in depth causal analysis is transferred to independent social frameworks principally Lipsky’s street level bureaucracy and the concepts of bureaucratic violence to thoroughly explain why these patterns persist and how localized administrative discretion transforms into systemic structural exclusion.

### 2.5 Triangulation procedures

To ensure interpretive depth, minimize confirmation bias, and build a cohesive qualitative verification network, this research implements a multi perspective qualitative source triangulation procedure. This protocol compares and contrasts the narratives shared by three distinct groups of social actors identified in the sampling matrix: first, the administrative and technocratic rationales provided by central state regulators; second, the lived experiences of the religious figures across the loci; and third, the specialized legal analysis provided by the expert analyst. By comparing interpretations across these diverse institutional boundaries, the triangulation protocol mitigates context specific bias, verifies the operational reality of administrative frictions, and establishes a balanced, dialogical empirical baseline that accurately reflects the localized complexities of religious governance.

### 2.6 Researcher positionality

In qualitative critical research, the researcher is not a neutral, detached observer. The researcher in this study holds a dual positionality as both an academic outsider and an insider actively engaged within the network of religious organizations in Indonesia. This insider status provides a significant methodological advantage, particularly in building rapport and facilitating access to grassroots communities during fieldwork. Nevertheless, the researcher is fully aware that this emotional proximity and shared sosioreligious identity can potentially inject confirmation bias, where informants might incline toward sharing victimhood narratives or the researcher might unconsciously direct data interpretation subjectively. To maintain objectivity, neutrality, and scientific balance, the researcher employed strict mitigation measures: first, the fieldwork involved independent third party local intermediaries to act as objective cultural bridges; second, the researcher adhered to strict non judgmental interview techniques without intervening with personal opinions.

## 3 Results and discussion

### 3.1 Results

#### 3.1.1 Spatial and socio legal trajectories across two decades: a historical perspective on religious governance

At the elite level, PBM 2006 is framed through technocratic language that projects a highly harmonious implementation, reducing potential conflicts to mere formal administrative procedures. However, field findings in this study confirm that this elite impression of harmony stands in stark contrast to the grassroots reality, where the regulation is frequently contextualized in a restrictive manner by local actors, thereby transforming into a mechanism of structural obstruction that drives societal polarization. This fractured dynamic is corroborated by data from Indonesian advocacy organizations, such as the SETARA Institute, which recorded a surge in intolerant acts at the end of 2024, with house of worship rejection remaining the dominant case. While the Commission for the Disappeared and Victims of Violence (KontraS) (2025) documented numerous religious freedom violations specifically involving worship prohibitions and physical permit barriers, for minority communities, this regulation ultimately acts not as an instrument of order, but as a discursive bottleneck that effectively obstructs religious expression (Ridwan, 2026). Finally, the National Commission on Human Rights (Komisi Nasional Hak Asasi Manusia Republik Indonesia, 2020), the national human rights institution in Indonesia, also assesses that the regulation facilitates the dominance of majority groups, leading to a formal recommendation for its revision into a law level regulation to better protect constitutional rights.

To trace how these administrative contradictions and discursive bottlenecks crystallize at the grassroots level, this study maps the empirical field developments linearly and chronologically across three distinct temporal periods spanning the last two decades. This periodic classification reveals profound structural shifts, beginning with an initial phase of spontaneous, locally driven dispute resolutions following the political regime transition, moving toward the institutionalization of formalized bureaucratic administrative frameworks, and culminating in contemporary patterns of technocratic governance that are highly sensitive to digital ecosystems and public opinion landscapes.

#### 3.1.2 Period I: early transition and institutional restructuring of the civic public sphere (2001–2006)

The first period (2001–2006) represents an early post New Order transition and the dawn of the Reformasi era, characterized by intense institutional restructuring wherein emerging state apparatuses frequently encountered dynamic community activities regarding the utilization of civic space. This study captures the initial manifestations of these local dynamics through the personal narrative of Pastor David Widhi Prasetya of the Christian Church of Java (Gereja Kristen Jawa), reflecting on a transitional experience in 2001 in Somawangi Village, Banjarnegara, Central Java. Despite completely satisfying the legal requirements for land tenure, the community’s plan to utilize the church building faced prolonged delays due to oppositional mobilization from certain local groups influenced by external non local networks. Under intense external pressure, the local community eventually agreed to an administrative compromise that restricted the building’s usage for formal worship services to preserve immediate sociopolitical stability. Sosiologically, this case serves as an early portrait of how spatial frictions during this era were predominantly managed through *ad hoc*, security driven containment strategies due to the absence of standardized, rigid licensing mechanisms.

#### 3.1.3 Period II: formal codification and the bureaucratization of religious governance (2006–2014)

This fluid transition subsequently prompted the second phase (2006–2014), which established formal codification through the enactment of the Joint Ministerial Decree (Peraturan Bersama Menteri/PBM) Numbers 9 and 8 of 2006. At the elite level, this policy was framed through technocratic discourse projecting orderly, systematized execution and encapsulating potential disputes within formal administrative channels. According to KH. Abdul Manan Ghani, former Chairperson of the Mosque and Da’wah Division of PBNU and former Associate Secretary General of the Indonesian Ulema Council (MUI), the text was positioned as a collaborative technocratic consensus involving active participation from all major national religious councils. Within this paradigm, the text was operationalized as a protective social mechanism, wherein the demographically dominant majority was duty bound to safeguard minoritized communities to ensure that freedom of worship could be exercised proportionally and in accordance with democratic tenets.

To anchor the sociological context of this institutionalization phase, Dr. Muhammad Alfian Djafar, S. H. I., M. H., Secretary of the Legal and Human Rights Council of the Central Board of Muhammadiyah, provided a vital communal perspective on this shifting regulatory environment. Established in 1912, Muhammadiyah represents one of the largest and most historically significant modern socio religious organizations in Indonesia, wielding vast sociopolitical, educational, and religious influence through its institutional networks across the archipelago. Alfian explained that during the initial drafting phase, Muhammadiyah viewed PBM 2006 as a necessary “social fence.” The regulation was originally intended as a structural compromise to preempt large scale horizontal instability at the grassroots level, drawing hard lessons from historical conflicts such as those in Poso, Central Sulawesi.

However, evaluating its operational realities after nearly two decades of implementation, Alfian concluded that the decree contains fundamental structural deficiencies. Specifically, the rigid demands for numerical thresholds and neighborhood consensus impose an exhausting administrative burden on minoritized groups. This institutional evaluation crystallizes a pivotal sociological shift: obstacles surrounding house of worship permits have now evolved beyond binary interfaith friction, increasingly complicating internal group dynamics and eroding social cohesion within the dominant majority itself.

These institutional challenges manifested concretely on the ground through the testimony of Pastor Palti Panjaitan regarding the HKBP Filadelfia congregation in Bekasi, West Coast Java. Prior to the escalation of administrative resistance, the congregation had actively cultivated organic social capital and civic embeddedness through grassroots humanitarian and community welfare initiatives. These initiatives included funding local community infrastructure, supporting public education centers, repairing village roads, and providing social safety nets for impoverished neighbors. Despite these deeply embedded emancipatory efforts, the community’s right to space was ultimately constrained by local administrative discretion designed to manage populus political anxieties, coupled with targeted mobilization from external non local actors.

#### 3.1.4 Period III: contemporary technocratic dynamics and grassroots coping strategies (2014–2026)

This trajectory advanced into the third period (2014–2026), defined by the absolute formalization of rigid administrative metrics, where technocratic mandates for official quotas and community consensus directly alter traditional layers of local coexistence. Field findings demonstrate that at the grassroots level, these bureaucratic provisions are implemented restrictively by local actors, fundamentally reshaping daily civic interactions. This period is marked by the manifestation of the previously identified sociological shift, wherein intra group fractures emerge within the dominant majority itself.

In another geographic context, administrative and structural containment also manifests as intra religious friction, which peaked between 2021 and 2022, as evidenced by the systematic resistance faced by the Muhammadiyah organization in Banyuwangi. The institutional development of this Islamic modernist movement has been severely hindered by localized majoritarian pressures, which include the vandalism of organizational and educational signboards in Tampo, Cluring, and Glenmore, as well as the forced halting of the Al Furqon Mosque construction in Sraten due to contested building permits (IMB) and unfavorable recommendations from the FKUB. Furthermore, this resistance escalates into overt social hostility and physical threats, ranging from the weaponization of land waqf disputes in Kalibaru, violent blockades against Friday prayers in Blimbingsari, to the obstruction of inclusive social projects, such as the initial rejection of a boarding school for children with special needs (Adawiyah and Jatmiko, 2024).

The conflict surrounding the At Taqwa Mosque in Sangso Village, Samalanga, Aceh, which spanned from 2015 to 2022, was triggered by the opposition of the majority traditionalist *pesantren* group toward the construction plan of a new mosque by Muhammadiyah members, following the takeover of the previous mosque’s management by traditionalists. This tension shifted into a struggle for power influence and social capital through the dissemination of negative stereotypes, where the protest and mass mobilization were uniquely driven by people from outside the village. On the other hand, the local Muhammadiyah Branch Board remained steadfast on their legal rights since there was no official religious fatwa banning their teachings, leaving the local government’s mediation an entangled thread that was difficult to resolve due to the clashing interests between these groups (Mugni et al., 2022).

While the cases in Banyuwangi and Samalanga illustrate intra religious frictions, identical patterns of administrative containment within inter religious, majority minority demographic landscapes are clearly reflected in the dispute over the Nur Musafir Mosque in Batuplat, Kupang City, which lasted from 2011 to 2016. This case raises a crucial question regarding whether the religious militancy of the majority group consistently dominates public policy, particularly concerning the fulfillment of the right to establish houses of worship for minorities (Hutapea, 2020). Furthermore, similar structural barriers are also experienced by the city’s Buddhist community, whose institutional struggle for a permanent place of worship spanned from 2015 to 2021, constituting the smallest religious minority in Kupang. As the most numerically marginal group, they have endured a protracted institutional struggle to establish a permanent place of worship (Hutapea, 2020). A similar phenomenon occurs among urban communities in Bali, where the establishment of formal houses of worship faces intricate structural and jurisdictional resistance across multiple regions. In Buleleng, the construction of the Jabal Nur Mosque was halted due to overlapping administrative claims between the local state office and the traditional village. This majoritarian barrier is further evidenced in Denpasar and Badung, where long standing prayer rooms, namely the al Syafi’iyyah and al Qari musalas, faced severe local protests and structural pushbacks from the resident majority regarding neighborhood consensus and environmental permits (Fahham, 2018).

These incidents reveal that when local opposition emerges, front line bureaucrats consistently prioritize pragmatic, security centric mediation to preserve short term order, thereby deferring definitive administrative resolutions. Discursive catchphrases such as “social harmony” function with strategic ambiguity, expanding front line diskretion to accommodate populist demands to stabilize local political equilibria (Cowan, 2022). By formalizing numerical baselines, this framework conditions inclusive inter communal relationships into zero sum calculations sensitive to identity politics (Bernal Triviño et al., 2026). This tension sparks critical debates regarding the alignment of state texts with Habermasian communicative rationality, which demands uncoerced, egalitarian public dialogue (Habermas, 1985; Kellner, 2014).

Faced with contemporary contemporary barriers, minoritized community leaders have engineered micro level social adaptation strategies to navigate this technocratic environment. This resilience is exemplified by Ws. Budi Rohadi, a Confucian cleric and Secretary of the Hok Tek Bio Tri Dharma Temple (TITD) in Purwokerto, and Yulia Susanti Sanjaya, Secretary of the Karuna Maitreya Vihara in Cilacap, Central Java. Drawing on decades of institutional experience, these leaders have developed proactive communication networks as an essential instrument of survival. As Sanjaya explains, the community in Cilacap prioritizes deep cultural acculturation and organic neighborhood integration, ensuring that the congregation blends into the social fabric long before initiating formal physical consolidation of their buildings. This sentiment is echoed by Slamet Rahardjo, Chairperson of the Parisada Hindu Dharma Indonesia (PHDI) in Banyumas Regency, who underscores that genuine inter village mutual aid and a shared sense of cultural fraternity serve as powerful tools for structural alignment.

Through the continuous execution of secular, cross communal humanitarian actions such as annual blood drives, public health screenings, local environmental cleanups, and community sports events minoritized groups successfully cultivate organic neighborhood legitimacy. Sanjaya notes that under this framework, daily interaction proceeds harmoniously, provided that external political actors do not intervene to disrupt these traditional layers of coexistence. Concurrently, Budi Rohadi emphasizes that maintaining local peace in contemporary urban landscapes relies heavily on preemptive and proactive communicative networks. Rohadi observes that contemporary permit gridlocks frequently stem from the informal use of commercial spaces (such as shophouses) prior to completing comprehensive administrative negotiations, which often generates local anxiety as congregation sizes expand.

Furthermore, this contemporary configuration uncovers that local administrative and social resistance is rarely driven by pure theological incompatibility. Rather, it is profoundly structured by underlying material factors, such as unresolved familial land inheritance disputes or municipal zoning non compliance when converting commercial spaces, where procedural friction erupts precisely because physical space is occupied before achieving communicative alignment with local residents.

#### 3.1.5 Deconstruction of regulatory clauses: lexical design and mechanisms of administrative gatekeeping

The functional transformation of regulatory instruments from tools of orderly governance into formal markers of symbolic exclusion highlights the linguistic complexity of bureaucratic texts in Indonesia. This study deploys Fairclough’s three dimensional Critical Discourse Analysis (CDA) framework as an organizational architecture to classify empirical findings across three interlocking registers: the textual dimension (lexical choices and sentence ambiguity), discursive practice (the production and consumption of policy texts), and sociocultural practice (the broader structural context of local society). Through this structural mapping, PBM 2006 is analyzed as a site of negotiated meaning that directly governs majority minority dynamics. Field findings confirm that structural asymmetries and procedural obstructions are systematically institutionalized through the linguistic design of these bureaucratic texts, specifically through the operationalization of Articles 8, 9, 13, and 14 of PBM 2006.

##### 3.1.5.1 Descriptive characteristics and implementation of article 8 (FKUB recommendations)

The institutionalization of this spatial gatekeeping is codified in Article 8, which establishes the Inter Religious Harmony Forum (Forum Kerukunan Umat Beragama/FKUB) at the municipal and regency levels. According to the original text of paragraph 1, the FKUB is tasked with facilitating inter religious harmony within its jurisdiction and issuing formal written recommendations for house of worship construction, while paragraph 2 mandates its function as a mediator in localized religious disputes.

The lexical architecture of Article 8 illustrates how functional ambiguity operates within front line governance. The deployment of verbs like “to facilitate” and “to provide recommendations” normatively frames the municipal FKUB as a consultative, advisory body designed to foster participatory, cross communal dialogue. However, field data demonstrates that in practice, these recommendations function as a de facto administrative veto. The gap between text and field execution is driven by the skewed proportional composition of regional FKUB boards. Field findings reveal that when consensus via musyawarah fails, final decisions default to a formal voting mechanism. Sosiologically, this majoritarian voting protocol places minoritized groups at an immediate numerical disadvantage. While the text does not explicitly mandate majoritarian dominance, board seats are allocated proportionally based on the local demographic density of each religious group, ensuring that representatives of the locally dominant majority automatically command a supermajority of seats. The consequence of this numerical democracy is that final outcomes directly reflect majoritarian preferences, transforming the forum from an inclusive, deliberative public sphere into a technocratic gatekeeper.

##### 3.1.5.2 Descriptive characteristics and implementation of article 9 (gubernatorial and mayoral discretion)

This administrative formalization is reinforced by executive discretionary powers codified under Article 9 regarding the role of Regional Heads. Based on the original text of paragraph 1, the regional executive (Regent/Mayor) holds the formal authority to issue building permits for houses of worship, “taking into tight consideration” the recommendations of the FKUB and the input of the local community. Paragraph 2 expands this by allowing the executive to seek further guidance from relevant bodies prior to making a final determination. In practice, regional heads wield expansive discretionary leeway, where final executive actions are heavily conditioned by immediate calculations of local sociopolitical stability, frequently resulting in indefinite administrative delays for minoritized pemohon despite their compliance with baseline requirements.

Based on the empirical insights provided by Father Martinus Ngarlan, Chairperson of the Commission for Inter Religious and Interfaith Relations (HAK) of the Purwokerto Diocese, field operations consistently encounter significant procedural stagnation across several localized jurisdictions. Within the framework of local governance, administrative delays or heightened social objections often prolong the verification process, even after applicants have satisfied formal statutory requirements. Father Ngarlan implies that this friction reflects an institutional challenge at the regional level, where administrative actors frequently prioritize immediate social stability, the mitigation of sociopolitical risks, and the protection of local political careers over formal clearances. Consequently, the progression of technically compliant zoning permits becomes heavily contingent upon the preservation of localized social equilibrium and the bureaucratic aversion to electoral backlash.

This structural asymmetry is embedded within the lexical choice of Article 9, where the verb “to consider” grants the regional executive boundless interpretive autonomy. Within the architecture of governance, this phrasing opens a vacuum for the personalization of executive power over formal statutory certainty, subjecting administrative processes to majoritarian pressure or electoral calculations. Regional executives face intense political communication dilemmas regarding their localized electoral bases if they issue permits opposed by dominant local networks. Field findings demonstrate that “to consider” is routinely translated into a mandate for indefinite deferral, cementing a public perception that administrative rights are negotiable commodities contingent upon security calculations. When localized friction erupts, regional authorities frequently default to a passive stance to secure short term peace, highlighting a sociological need to restructure executive powers around principles of strict transparency and non negotiable compliance.

##### 3.1.5.3 Descriptive characteristics and implementation of article 13 (the “real need” clause)

Localized administrative friction is further intensified by the subjective performance indicators codified under Article 13. Paragraph 1 stipulates that a house of worship can only be established if there is a demonstrated “real and earnest need” among the adherents within that specific village or kelurahan. Paragraph 2 provides a cascading clause stating that if the numerical threshold cannot be met at the village level, the demographic composition may be evaluated at the sub district, municipal, or provincial levels. In practice, this clause imposes a punishing spatial burden on demographically dispersed or small populations, forcing them to undergo exhausting legal gymnastics to prove the spiritual urgency of their facilities to local bureaucratic actors.

The discursive critical register of Article 13 centers on the phrasing “real and earnest need,” an inherently subjective lexical choice lacking objective, standardized metrics. This lack of precision complicates formal compliance for minoritized groups. Field findings document instances where minoritized citizens are forced to travel vast distances across administrative boundaries to access basic worship facilities due to their failure to meet the localized village quota. This highlights a structural contradiction where constitutional freedoms are constrained by geographic and procedural barriers. Furthermore, the provision allowing demographic evaluation to cascade to higher administrative levels (from village to province) discursively frames a fundamental right as a function of demographic mass. This reproduces a majoritarian logic that subordinates universal rights to local population density, reinforcing the sociological urgency to replace population metrics with objective urban planning assessments independent of group size.

##### 3.1.5.4 Descriptive characteristics and implementation of article 14 (numerical user and supporter quotas)

These spatial planning constraints are directly tied to the quantitative metrics mandated by Article 14, paragraph 2. This clause dictates that any application for a house of worship must secure a certified list of at least 90 actual users and verified signatures of support from at least 60 local neighborhood residents, formally endorsed by the village head (lurah/kepala desa), alongside the mandatory FKUB clearance. These quantitative thresholds are reiterated across subsequent paragraphs as the central prerequisites for legal spatial existence, functioning on the ground as a formidable barrier for minoritized populations across various regions.

Conceptually, the requirement for 60 local neighborhood signatures can be analyzed from an administrative governance perspective as a form of discriminatory procedural barrier. This structural framing inherently equates the construction of a spiritual facility with an environmental or industrial disruption that requires immediate social clearance from the surrounding neighborhood, rather than positioning it as a constitutional right that must be vertically facilitated by the state. Field findings reveal that the process of gathering verified identity cards (KTP) from local neighbors consistently triggers administrative labyrinths and horizontal friction, as local supporters face intense majoritarian social pressure or intimidation. From a policy perspective, these systemic barriers highlight the necessity of shifting religious governance from majoritarian veto permissions toward an objective, notification based registration system focused strictly on neutral municipal zoning codes and non discriminatory urban planning standards.

These These domestic dynamics regarding procedural governance position Indonesia’s regulatory framework within international human rights debates, particularly concerning compliance with the ICCPR. When international indices highlight government restriction parameters well above global averages, these metrics are interpreted within sociocultural practices as empirical indicators of systemic majoritarian gatekeeping. Indeed, paradoxes arise when global data reveal another side of Indonesia’s reality; Pew Research notes that Indonesia is categorised as very high in the Government Restrictions Index, with a score of 7.9, and 4.7 in the Social Hostilities Index in 2022. This empirical evidence underscores the fact that religious life in Indonesia is governed by strict regulation, while horizontal tensions persist (Ginting et al., 2026). In this context, routine compliance demands and front line discretion operate as containment mechanisms within the civic public sphere. The lexical choices within these bureaucratic texts codify a form of administrative stratification, validating majoritarian preferences through state mechanisms and directly linking daily public administration with the spatial containment of minoritized groups at the local level.

### 3.2 Discussion

#### 3.2.1 Front line bureaucratic dilemmas and executive discretion in local governance

The empirical field findings reveal a stark contradiction between the inclusive, consensus driven elite paradigms projected by KH. Abdul Manan Ghani and Dr. Muhammad Alfian Djafar, and the restrictive, security centric compliance executed by street level administrative actors. This operational disconnect is anchored in deep rooted local dynamics, spanning from Pastor David Widhi Prasetya’s narrative of forced administrative compromise in Somawangi Village in 2001, to the contemporary insights provided by Father Martinus Ngarlan regarding systemic delays and heightened social friction within the specific regional jurisdictions under his pastoral observation. In these instances, front line bureaucrats consistently default to a passive stance and withhold executive signatures under majoritarian pressure to insulate their political careers from risk. These combined field insights demonstrate that street level administrative choices are dictated by calculated career survival rather than formal statutory certainty, illustrating the acute operational tensions within local religious governance.

To interpret these institutional dynamics, the application of Michael Lipsky’s seminal framework on street level bureaucracy becomes paramount (Lipsky, 1980). Administrative actors at the grassroots level are structurally positioned within a complex role contradiction: they are mandated to enforce impersonal national regulations, yet are held directly accountable for preserving immediate localized sociopolitical stability. When waves of majoritarian populist mobilization exert pressure on regional state nodes, front line bureaucrats consistently deploy their discretionary leverage to enact pragmatic accommodations, thereby insulating their careers from political risk. This operational reality substantiates Aragon's (2022) socio legal analysis, which demonstrates how localized religious expressions are systematically altered when intersecting with modernist bureaucratic institutions that reflexively align with dominant numerical aggregates.

This structural instability and the systemic hollowing of regulatory predictability are fundamentally rooted in the precarious constitutional hierarchy of the joint decree itself. Formally, the legal position of PBM 2006 is compromised because this specific regulatory instrument is not explicitly recognized within the statutory hierarchy of Law No. 12/2011 on the Formulation of Laws and Regulations. Even when evaluated under Article 8 of the Law, which accommodates various non hierarchical ministerial regulations, the text remains silent on joint instruments. This omission confirms that the Joint Ministerial Decree fails to reflect formal legality, as its validity lacks both a clear statutory source and proper issuing authority (Junaidi, 2013). Consequently, despite its functional utility in facilitating inter ministerial coordination, PBM 2006 operates within a state of formal statutory precarity. As evaluated by Prof. Dr. Abdul Aziz Nasihuddin, an expert in Administrative Law, this statutory omission means that to guarantee robust legal certainty and protect minority spatial rights from majoritarian capturing, the regulatory status of these provisions should be upgraded to a Presidential Regulation or a statute level Law. Nasihuddin further contends that while the decree historically functioned as a temporary transitional instrument to address localized legal vacuums on the ground, its long term statutory precarity leaves the protection of front line bureaucrats unestablished. This structural vulnerability underscores a deeper systemic problem: the lack of a standardized legislative baseline that defines the legitimate boundaries of state intervention. Under the Indonesian constitutional design, it is firmly established that in exercising their rights and freedoms, every citizen is obliged to comply with the restrictions stipulated by law as referred to in Article 28J of the 1945 Constitution (Rumadi, 2020). Consequently, to effectively balance these fundamental rights and obligations and provide administrative actors with clear statutory protection, future legislative development must be enriched through international comparative studies with countries holding minoritized Muslim cultures. This comparative approach is essential to map out the accommodative thresholds of state governance in Indonesia, ensuring that any enforced statutory limitations remain democratic, proportional, and legally sound.

This administrative accommodation conditions a compromised democratic environment, where state passivity in the face of majoritarian pressure transforms routine regulatory enforcement into a mechanism of structural obstruction that directly marginalizes demographically minoritized communities (Qodir and Hefner, 2024). This order is not driven by top down autocratic decrees; rather, it operates via the daily conditioning of local state actors. Lacking robust institutional insulation from regional political networks, front line bureaucrats convert formal legal parameters into conflict mitigation tools. Phenomena such as administrative stonewalling, the calculated deferral of recommendations, or the intentional shelving of applications must be understood through Michael Lipsky’s seminal axiom on front line statecraft. Lipsky argues that “the decisions of street level bureaucrats, the routines they establish, and the devices they invent to cope with uncertainties and work pressures, effectively become the public policies they carry out” (Lipsky, 1980). This institutionalization of administrative delay forms an unwritten de facto policy precedent, establishing that formal statutory certainty is routinely subordinated to the sociopolitical comfort of local state managers.

Consequently, this institutionalization of administrative metrics conditions how contemporary societies are governed, where the rigid demands for official numbers and community consensus effectively disrupt traditional layers of local coexistence. This friction is exacerbated by misaligned institutional performance indicators, forcing front line bureaucrats to implement indirect rationing of services to conserve administrative and political energy. To manage this moral dilemma, local actors develop psychological coping mechanisms rooted in a mentality of bureaucratic abdication deeming their statutory obligations fully discharged by shifting the burden of decision making entirely onto localized communal forums, regardless of statutory finality. This environment breeds a pattern of social categorization, segregating citizens into “cooperative” networks versus those pathologized as “security risks” (blaming the victim). Within this fragmented public sphere, even when minority groups actively cultivate cultural citizenship and interfaith cohesion through inclusive humanitarian actions, their emancipatory efforts are routinely suppressed by exclusive majoritarian networks that dominate local socio legal structures (Qodir and Hefner, 2024).

#### 3.2.2 Bureaucratic violence: procedural mechanisms, policy ambiguity, and the erasure of civic space

The persistent licensing delays, targeted local mobilizations, and the structural contraction of civic spaces documented across the field demonstrate how technical metrics are operationalized into administrative filters, even when minoritized groups establish deep neighborhood legitimacy. This pattern of systemic spatial restriction is concretely manifested through the administrative challenges against Pastor Palti Panjaitan’s HKBP Filadelfia congregation in Bekasi, the forced dismantling of Muhammadiyah institutional signage in Banyuwangi, the persistent permit impediments surrounding the At Taqwa Mosque in Samalanga, and the retroactive threshold challenges facing the Nur Musafir Mosque in Kupang, the Jabal Nur Mosque in Badung, and the Al Syafi’iyyah and Al Qari musalas in Denpasar. Confronted with these mathematical gatekeeping barriers, minoritized leaders like Ws. Budi Rohadi in Purwokerto, Yulia Susanti Sanjaya in Cilacap, and Slamet Rahardjo in Banyumas engineer micro level adaptation strategies, utilizing cultural acculturation and organic communication networks as vital survival mechanisms.

These empirical realities underscore that long term spatial restriction operates not through overt physical coercion, but through the routine deployment of formal legal protocols. Consequently, these chronic administrative delays documented at the grassroots level must be substantively reframed as a structural administrative constraint, wherein systemic exclusion functions through the silent, technocratic utilization of formal state apparatuses. Within the global landscape of social stratification, this structural neutralization of minoritized entities frequently operates through the deployment of bureaucratic obstruction (Heckert, 2020), wherein technocratic state protocols are systematically utilized to enforce long term spatial containment (Rhoads et al., 2025). The deployment of quantitative metrics such as user thresholds and neighborhood supporter quotas functions as a highly restrictive mechanism of spatial gatekeeping. For communities with small or geographically dispersed populations, satisfying these mathematical mandates is structurally unfeasible, thereby enforcing an institutionalized contraction that systematically denies minoritized groups formal spatial recognition and access to the civic public sphere.

Comparatively, this technocratic closure of civic space exhibits a powerful theoretical portability, directly resonating with contemporary socio legal dynamics globally. Under Hindutva majoritarian nationalism in India, structural exclusion operates systematically through the mobilization of urban planning regulations, municipal zoning laws, and local spatial ordinances. At its core, this manifestation of Hindu nationalism under Modi’s Bharatiya Janata Party (BJP) is fundamentally about the attempted marginalization of a minority, namely, Muslim Indians, on the ground of their religion (Varshney and Staggs, 2024). Front line bureaucratic actors deploy routine administrative permits such as building safety certifications, land use verifications, or public nuisance codes as gatekeeping mechanisms to obstruct and enforce the spatial containment of minoritized communal facilities. These policies are discursively framed through neutral narratives of urban renewal, municipal order, and strict statutory compliance; however, in practice, they function as bureaucratic instruments to eradicate non dominant visibility from the public sphere. Deep analytical elaboration reveals that this majoritarian hegemony in India is crystallized through administrative protocols that deliberately complicate the historical verification of land titles for minoritized communities in urban enclaves, converting architectural compliance into an instrument of long term spatial containment that neutralizes civic emancipation. In this context, the weaponization of bureaucratic permits cannot be detached from a broader, dual track oppression where Hindu nationalism is using both legislative power and extralegal methods to subdue Muslims. Vigilante violence, condoned or supported by the state, has been on the rise since Modi and his party came to power. In short, elections are being used to create legislatures that pass anti Muslim laws, while street level vigilantism supports the formal politics of exclusion (Varshney and Staggs, 2024). Consequently, the technical micro politics of municipal zoning serve as the precise bureaucratic anchor that operationalizes this grand structural design of exclusion.

An identical pattern of spatial conditioning manifests within the landscape of right wing populism in Europe, where the technocratic closure of civic space is deeply intertwined with the politics of the rejection of immigrants. Constrained by international human rights frameworks that formally prohibit overt racial discrimination or forced physical expulsions, local administrative authorities influenced by right wing populist agendas pivot toward neutral, technocratic legal codes. They leverage municipal master plans, localized demographic density caps, and strict neighborhood architectural harmony codes to legally restrict the social spatialization of immigrant populations. Regulations are engineered through highly professionalized language, deploying narratives of heritage preservation, visual aesthetic conformity, or the prevention of local environmental congestion. Beneath this veneer of linguistic objectivity, licensing mechanisms are consistently converted into tools for the selective closure of civic space. This administrative maneuver is driven by the populist assertion that the presence of immigrants (Muslims) in Europe is inherently destabilizing and exacerbates public insecurity, (Haynes, 2020), which effectively justifies these technical interventions. Front line bureaucrats in these jurisdictions utilize their discretionary latitude to prolong document processing and quietly deny spatial permits to immigrant congregations when facing populist public pressure. Critically, this technocratic closure in Europe manifests an invisible exclusion wherein formal secular standards (such as acoustic pollution codes, commercial zoning transitions, or urban visual facade harmony) are operationalized as rigid administrative filters to screen out external groups, stripping minoritized migrant communities of baseline rights through the daily enforcement of seemingly impartial daily administrative rules.

This operational reality is reinforced by the linguistic strategies within policy texts that deliberately retain highly subjective clauses devoid of objective empirical baselines, creating a state of chronic administrative precarity for the applicant (Rhoads et al., 2025). Within this regulatory vacuum, a sociological cleft opens, inviting informal intervention by locally dominant communal networks. As Roggeband and Krizsán (2021) demonstrate, “closure is in fact a selective mechanism: governments attempt to reorganize civic space through a dual process of selective in and exclusion of civil society organizations.” Dominant communal networks exploit this lack of statutory precision as a tool of social pressure to capture local executive discretion, pulling public administration away from Habermas’s model of communicative rationality, which demands an uncoerced, egalitarian public sphere (Habermas, 1985; Kellner, 2014).

Within a majoritarian landscape, this technocratic closure of civic space is explicitly translated into the regulation of physical territory, turning the administrative permit process into a tool of spatial containment. When regional authorities opt for structural passivity or prioritize short term, populist accommodating compromises, their actions reflect a pattern of administrative backlash and state omission (Roggeband and Krizsán, 2021). This pattern of state abdication informally validates majoritarian overreach, allowing dominant networks to dictate the boundaries of physical territory and capture the civic public sphere. This enforcement of majoritarian order at the expense of substantive constitutional rights illuminates a core sociological challenge in balancing public order with inclusive, progressive governance (progress), a tension that aligns directly with Auguste Comte’s foundational concepts on social dynamics (Comte, 1855).

#### 3.2.3 Material conflicts and political economic inequality: rival sociological explanations across cases

The empirical data from the field uncovers that localized administrative and social resistance surrounding houses of worship is rarely driven by pure theological incompatibility, but is profoundly structured by underlying material anxieties and transaction oriented local governance. To avoid reductive cultural essentialism, this study integrates a competing alternative explanations framework by unearthing these underlying political economic inequalities. Beneath the highly visible identity debates that dominate the public surface, there consistently lies a structural clash of material interests, where surface level religious differences are frequently operationalized as a discursive veil to mobilize populist sentiment instantly within the public sphere. In reality, grassroots resistance is overwhelmingly driven by highly mundane, material anxieties, such as unresolved familial land inheritance disputes, localized commercial business competition, protectionist fears regarding the depreciation of neighboring property values, and municipal zoning non compliance when converting commercial shophouses.

As documented through the testimonies of Yulia Susanti Sanjaya and Ws. Budi Rohadi, contemporary permit delays frequently stem from these informal structural frictions, particularly when physical space is occupied before completing comprehensive administrative negotiations and achieving communicative alignment with local residents. Furthermore, these structural vulnerabilities are systematically exacerbated by transaction oriented, pragmatic conflict resolution models at the local level, where informal financial compensation packages transform procedural barriers into permanent avenues for institutionalized transactional incentives among particularistic local interests. Sociological analysis confirms that spatial frictions do not exist in a vacuum of identity politics, but are heavily conditioned by market driven calculations and material inequalities that position citizens’ rights within a cycle of continuous procedural renegotiation.

Furthermore, this political economic inequality is systematically exacerbated by transaction oriented, pragmatic conflict resolution models at the local level. Attempting to bypass administrative blockages through informal financial compensation packages does not resolve the structural zoning issues; rather, it transforms administrative vulnerabilities into permanent sources of rent seeking for specific local actors. When financial instruments are deployed as short term fixes to suppress localized social resistance without establishing substantive legal finality, the administrative rights of citizens become trapped in an unendurable cycle of transactional dependency at the grassroots level, rendering the permit process a permanent economic commodity exploited by local opportunistic networks. This reality highlights the profound challenges facing the domestic legal architecture in constructing standardized, intervention free regulatory enforcement. Unlike consolidated legal environments where municipal regulations are enforced uniformly to guarantee predictable civic coexistence, the local Indonesian administrative landscape remains deeply vulnerable to procedural renegotiation. This regulatory elasticity demonstrates how the accumulation of financial capital, elite bureaucratic connections, and majoritarian leverage operate within localized networks to dictate the final execution of formal policies.

The validity of this sociological argument is reinforced by the fact that spatial barriers are not unique to any single religious tradition. To fulfill the imperative for objective empirical data, this study explicitly integrates insights from secondary comparative literature on Indonesian interfaith relations (Qodir and Hefner, 2024). This extensive scholarship demonstrates that identical structural barriers are systematically encountered by the nationally dominant majority tradition when it exists as a localized numerical minority within regions dominated by alternative communal networks. When any denomination exists as a localized demographic minority it confronts an identical labyrinth of front line bureaucracy exhausting neighborhood supporter quotas and aggressive localized resistance.

Ultimately, these internal fractures within the same broad religious tradition transcend mere textual discrepancy, offering a vital sociological critique of the pathology of religious governance within the broader discourse of challenges to democratic governance and sustainability. When the coordinates of the public sphere are governed through rigid, majoritarian biased administrative metrics, the law ceases to function as an instrument of social integration; instead, it is converted into a highly contested arena of spatial hegemony. Local factions within the dominant tradition are forced to compete against one another for claims of theological authenticity, formal state validation, and administrative permits over territory. Consequently, regulations originally justified as tools for social harmony systematically generate internal competition that erodes the social fabric of the dominant community itself, directly threatening the long term sustainability of peace at the grassroots level. Localized disputes over organizational attributes and institutional signage prove that rigid proceduralism without substantive rights acts as a divisive vector that fractures traditional networks of solidarity. This patterns seals a critical sociological conclusion aligned with multi case secondary literature: majoritarian biased bureaucratic enforcement not only restricts spatial access for minoritized populations, but consistently reproduces deep structural fractures within the dominant majority itself.

## 4 Conclusion

This two decade study on religious governance in Indonesia demonstrates that the institutionalization of rigid administrative metrics preserves structural asymmetries, thereby connecting localized spatial disputes directly with broader macro sociological patterns of social stratification, institutional inequality, and systemic marginalization. Analyzed through the lens of street level bureaucracy, localized licensing barriers reflect a systemic manifestation of structural administrative constraint and spatial exclusion operating through formal legal protocols to contract civic space, where the lack of precise baselines leaves the regulatory framework highly vulnerable to localized capturing and underlying material conflicts. This reality solidifies a crucial sociological conclusion that the asymmetric distribution of spatial rights originates not from theological doctrines, but from modernist bureaucratic pathologies and structural power dynamics left open to renegotiation to accommodate majoritarian political equilibria. Comparatively, this phenomenon of administrative constraint possesses strong theoretical portability, as it simultaneously echoes global landscapes of spatial containment and minoritized vulnerability, such as the strategic deployment of urban planning and zoning regulations under Hindu nationalism in India and the selective exclusion of civic space driven by right wing populism in Europe. By advancing the concept of the state’s juridical resonance, this study unearths how the structural ambiguity of policy texts from above does not operate in a vacuum; rather, it actively conditions a micro level marketplace of informal transactional incentives, grassroots anxieties, and particularistic patron client networks that commodifies spatial recognition. Ultimately, dismantling these asymmetric boundaries requires an institutional reconstruction that replaces discretionary local consensus with depoliticized and highly rationalized zoning frameworks. Stripping localized networks of the regulatory elasticity they exploit to capture state discretion is paramount to neutralizing uneven patterns of spatial citizenship, reshaping the institutional conditions of local coexistence, and cultivating a transformative model of inclusive cultural citizenship.

## Data Availability

The original contributions presented in the study are included in the article/supplementary material, further inquiries can be directed to the corresponding author.
